# Serum hepcidin concentration is lower in advanced stages of sporadic colorectal cancer

**DOI:** 10.11613/BM.2025.030703

**Published:** 2025-10-15

**Authors:** Tara Rolić, Sanja Mandić, Mazyar Yazdani, Marina Ferenac Kiš, Sonia Distante, Ines Banjari

**Affiliations:** 1 Faculty of Medicine, University of Osijek, Osijek, Croatia; 2 Institute of Clinical Laboratory Diagnostics, Osijek University Hospital Centre, Osijek, Croatia; 3 Medical-biochemistry laboratory, Policlinic LabPlus, Osijek, Croatia; 4 Department of Medical Biochemistry, Oslo University Hospital, Oslo, Norway; 5 Clinical Institute for Transfusion Medicine, Osijek University Hospital Centre, Osijek, Croatia; 6 Faculty of Food Technology Osijek, University of Osijek, Osijek, Croatia

**Keywords:** anemia, colorectal cancer, hepcidin, homeostasis, iron

## Abstract

**Introduction:**

Hepcidin (Hep), a key regulatory hormone of iron (Fe) homeostasis, governs its absorption and storage, and is influenced by inflammation and Fe status. This study investigated serum Hep concentrations and their associations with Fe markers and inflammation in patients with sporadic colorectal cancer (CRC).

**Materials and methods:**

We compared serum concentrations of Hep, Fe, unsaturated and total iron binding capacity, transferrin, transferrin saturation, ferritin, C-reactive protein (CRP), interleukin-6 (IL-6) and tumor markers in 82 CRC patients and 58 controls. Statistically significant differences were tested using the Mann-Whitney U test and Student’s t test. Additionally, Hep were analyzed according to tumor stage. Colorectal cancer was confirmed histopathologically after colonoscopy with biopsy (TNM staging).

**Results:**

Colorectal cancer patients exhibited significantly lower Hep concentrations than controls (8.1 *vs*. 19.7 ng/mL, P = 0.020). Ferritin was also lower in CRC (109 *vs*. 250 µg/L, P = 0.002). Hepcidin showed the strongest positive correlation with ferritin in CRC. Inflammatory markers (CRP and IL-6) correlated moderately to weakly with hepcidin in both groups (controls: rho = 0.52 (P < 0.001); CRC: rho = 0.26 (P = 0.022) for CRP and CRC: rho = 0.30 (P = 0.033) for IL-6). Notably, Hep concentrations were lower in patients with advanced tumor stage (T0 *vs*. T3, P = 0.043).

**Conclusion:**

These findings suggest that CRC is associated with lower hepcidin and ferritin concentrations, potentially reflecting complex and cancer-specific dysregulation in Fe metabolism beyond inflammation alone.

## Graphical abstract

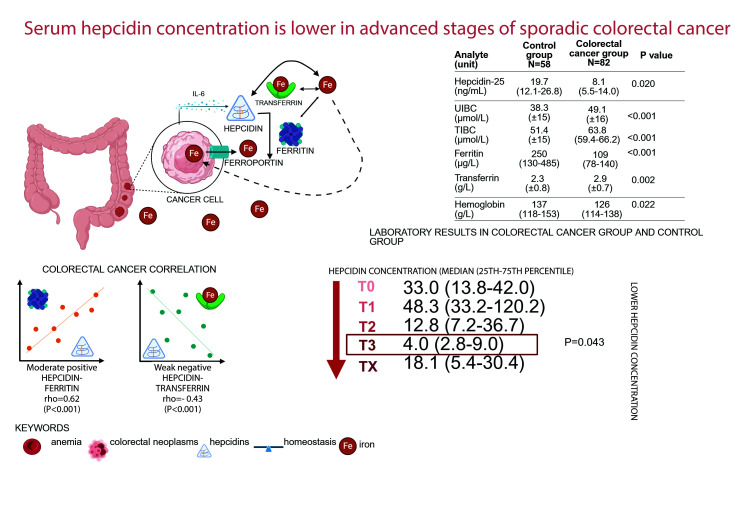

## Highlights

• CRC patients show lower hepcidin concentration than controls

• Hepcidin is moderately linked to ferritin, reflecting iron dysregulation in colorectal cancer

• Colorectal cancer patients have lower ferritin than controls

• Hepcidin is lower in advanced tumor stage (T3 *vs*. T0)

• C-reactive protein and interleukin-6 correlate weakly with hepcidin, suggesting other tumor effects

## Introduction

Iron metabolism plays a crucial role in health and disease, requiring tight regulation to avoid iron deficiency or overload. Excess iron promotes oxidative stress, while deficiency impairs cellular function (*1*). Hepcidin (Hep), a liver-derived peptide hormone, is the master regulator of systemic iron homeostasis (*2*). It inhibits iron export by binding to ferroportin, the only known iron exporter, leading to its degradation and lower serum iron concentrations (*2*, *3*). Hepcidin expression is influenced by iron stores, erythropoietic demand, and inflammation (*4*). It is often elevated in chronic inflammation and certain cancers, contributing to anemia of inflammation (*5*). Anemia of chronic disease (or anemia of inflammation) is increasingly prevalent in chronic conditions where reduced iron recycling from macrophages is observed (*6*). Inflammatory response and the release of inflammation-induced cytokines, such as interleukin-6 (IL-6), triggers Hep synthesis. Hepcidin, upregulated by IL-6, binds to ferroportin and limits iron availability in the plasma, leading to hypoferremia and anemia of chronic disease or anemia of inflammation (*7*). However, different measuring methods, whether mass spectrometry or immunochemical principles, have produced varying results and challenges in methodological standardization, complicating clinical interpretation (*8*).

In colorectal cancer (CRC), disturbances in iron metabolism are common and often manifest as anemia of inflammation or iron-deficiency anemia (*9*). Previous studies have shown conflicting results regarding Hep concentrations in CRC, presenting a more complex scenario, where iron loss by bleeding, inflammation, and tumor-associated iron demand coexist. Evidence suggests both, upregulation due to inflammation and downregulation related to tumor-specific iron demands. While altered iron biomarkers and anemia are common in CRC, the role of Hep remains unclear, with conflicting reports on its expression and clinical relevance (*9*, *10*). Overall, a better understanding of iron metabolism in cancer cells may also aid in the understanding of chemotherapy resistance (*11*).

Colorectal cancer is a major global health burden, and understanding its metabolic profile may aid early detection and treatment stratification (*12*, *13*). Mechanistically, unabsorbed dietary iron that remains in the gastrointestinal tract may have potentially carcinogenic effect (*14*). Despite its central role in iron regulation, Hep has not been extensively studied as a potential biomarker in sporadic, non-metastatic CRC.

The aim of this study was to investigate whether Hep serum concentrations are altered in a prospective cohort of CRC patients. We explored Hep correlation with iron metabolism, inflammation (C-reactive protein, CRP), tumor markers (carbohydrate antigen 19-9 (CA19-9) and carcinoembryonic antigen (CEA), tumor stage and anemia-related indices to better understand its role in CRC-related iron dysregulation and examined its potential as a biomarker of CRC. We hypothesized that Hep is dysregulated in CRC and reflects disease stage and systemic iron imbalance.

## Materials and methods

### Materials

A prospective cohort study was conducted in non-metastatic CRC patients and in a control group. Study participants were referred to the University Hospital Centre Osijek between August 2020 and August 2023. Blood sampling and completion of a food frequency questionnaire (FFQ) were performed after participants provided written informed consent, in accordance with ethical guidelines. Patients were recruited at the Department of Radiotherapy and Oncology, University Hospital Centre Osijek, following colonoscopy (the reference method for diagnosing CRC) and biopsy with histopathological confirmation of CRC. Tumor staging was classified as stage T0-T3 (N0-N3, M0) according to the tumor node metastasis (TNM) classification system of the American Joint Committee on Cancer: T0 – no histopathological evidence of a primary tumor (no tumor cells were detected in the sample), T1 – tumor invades the *submucosa*, T2 - tumor invades the *muscularis propria*, T3 - tumor invades through the *muscularis propria* into the pericolorectal tissues, Tx – primary tumor cannot be assessed due to insufficient information (*eg.*, poorly preserved tissue, incomplete biopsy, technical issues, or missing clinical/radiological data). Patients underwent colonoscopy due to clinical symptoms and/or positive guaiac fecal occult blood test (HemoGnost, BioGnost, Zagreb, Croatia) manually preformed at referring institutions or as part of the Croatian National CRC screening program.

Inclusion criteria for the CRC group included: confirmed diagnosis of sporadic colorectal cancer, male and female participants, aged between 40 and 80 years with a body mass index (BMI) of less than 35 kg/m^2^ and following a diet that included both animal- and plant-based foods. Patients with BMI > 35 kg/m^2^ were not excluded, as only one in the CRC group and two in the control group met this criterion. A structured FFQ was used to obtain information on diet type and supplement intake. These criteria ensured that the study population was both representative and homogenous, focusing on early-stage colorectal cancer patients with specific demographic and lifestyle characteristics. Exclusion criteria for the CRC group included the diagnosis of hereditary colorectal cancer (such as Lynch syndrome), colorectal cancer classified as an advanced stage (T4), additional cancer diagnoses, acromegaly, ulcerative colitis, Crohn’s disease, irritable bowel syndrome, parasitic or other infectious diseases, diverticulosis, or cystic fibrosis, transplant recipients, patients undergoing immunosuppressive therapy, or had type 1 or type 2 diabetes, active *Helicobacter pylori* infection, ongoing therapy with antacids, and the chronic use of non-steroid anti-inflammatory drugs (NSAIDs), participants receiving iron supplementation therapy or regularly used dietary supplements containing iron, vitamin B12, folic acid, vitamin D, or calcium, had a clinically significant weight loss (> 5% of body mass in the last 6 to 12 months), and a diet that excluded meat consumption. The control group inclusion criteria were male and female participants aged between 40 and 80 years, with a BMI less than 35 kg/m^2^ and adhering to a diet consisting of both animal- and plant-based foods. Exclusion criteria for the control group matched those of the colorectal cancer group. The control group was recruited during routine blood testing at the Institute of Clinical Laboratory Diagnostics, Osijek University Hospital Centre, meeting the inclusion and exclusion criteria.

This research was approved by the Ethical Committee of the University Hospital Centre Osijek, No. R2-8262/2020. and was conducted in accordance with the Declaration of Helsinki.

### Methods

All laboratory analyses were measured at the Institute of Clinical Laboratory Diagnostics, University Hospital Centre Osijek. Venipuncture was performed in the morning between 7:00 and 9:00 AM, while patients were fasting, in accordance with international recommendations (*15*). Blood was collected in K_2_EDTA coagulant tubes and anticoagulant-free tubes (Becton, Dickinson and Company, USA) which were centrifuged at 2000xg for 10 minutes. Afterwards, hemoglobin was measured from full blood sample, and serum samples were used for the determination of iron metabolism parameters including iron, unsaturated iron binding capacity (UIBC), transferrin, ferritin, CRP, CEA and CA19-9. Total iron binding capacity (TIBC) and transferrin saturation were calculated using the following formulas: TIBC = iron + UIBC; transferrin saturation (%Trf = iron / (TIBC x 100)). A Beckman Coulter AU680 analyzer (Beckman Coulter, Inc., Brea, USA) was used to measure iron and UIBC spectrophotometrically, while Trf, ferritin, and CRP were measured by immunoturbidimetry. Hepcidin concentrations were measured by enzyme-linked immunoassay (ELISA) using the HybridXL DRG analyzer (DRG Instruments GmbH, Marburg, Germany). The fully automated immunoassay has intra- (CVi) and interindividual (CVg) coefficients of variations provided by manufacturer of CVi = 2.55% and CVg = 13.68%, which were verified by our laboratory; linearity 1.67-81.00 ng/mL, limit of detection 1.67 ng/mL and limit of quantification 2.62 ng/mL with proposed reference ranges by manufacturer for males 1.67-49.75 ng/mL and females 1.67-56.70 ng/mL (*16*). Internal quality control at two levels provided by the manufacturer was used before the measurement of the samples and external quality control was not available. The immunoassay method on Roche Cobas e801 analyzer (Roche Diagnostics GmbH, Mannheim, Germany) was used to measure CEA, CA19-9 and IL-6. Hemoglobin concentrations were measured using Sysmex XN-2000 automatic analyzer (Sysmex Corporation, Kobe, Japan). While routine biomarkers were analyzed immediately upon sampling, aliquots for Hep determination were stored at - 20 ºC and analyzed after 6 months. According to the manufacturer, hepcidin remains stable under this storage conditions.

### Statistical analysis

Statistical analysis of data was performed using the MedCalc statistical program (MedCalc for Windows, version 12.4.0.0.; MedCalcSoftware, Mariakerke, Belgium). The D’Agostino-Pearson test was conducted to assess normality of data distribution. Results are presented as absolute counts for sex (male *vs.* female), age (range), and TNM stage for the colorectal cancer group; as median values and interquartile ranges (25th-75th percentile) for parameters following non-normal distribution (Fe, TIBC_CRC, ferritin, %Trf, CRP, CEA, CA19-9); and as means and standard deviations for parameters following a normal distribution (UIBC, transferrin, TIBC_CONTROL). Spearman’s rank correlation coefficient (rho) was used for non-normally distributed variables, and Pearson’s correlation coefficient (r) was used to for normally distributed variables. The Mann-Whitney U test and t-test was used for the comparison of Hep, CRP, iron, UIBC, TIBC, ferritin, transferrin and %Trf values in the CRC group *versus* the control group. The Kruskal-Wallis test was used to differentiate Hep concentrations in the CRC group across TNM stages. P-values less than 0.05 (P < 0.05) were considered statistically significant.

## Results

A total of 82 patients with sporadic, non-metastatic colorectal cancer were included in the study, comprising 22 females and 60 males. The median age of the participants was 65 years, ranging from 46 to 79 years. According to TNM staging, stage T3 was predominant (38/82), followed by an unknown stage (Tx) in 18/82 and less frequent cases of stage T2 (14/82). A total of 58 participants were included as the control group, consisting of 5 females and 53 males, with a median age of 58 years (range: 55-61).

The CRC group was significantly older (P < 0.001) and had a higher proportion of females (P = 0.013) compared to the control group.

Iron metabolism parameters are summarized in Table 1. Significant differences in Hep, ferritin, UIBC, TIBC, transferrin and hemoglobin concentrations between the CRC and control groups were found.

**Table 1:** Laboratory results of iron metabolism parameters including hepcidin, inflammation and tumor markers

**Analyte (unit)**	**Control group** **N = 58**	**CRC** **N = 82**	**P**
Hepcidin (ng/mL)	19.7 (12.1-26.8)	8.1 (5.5-14.0)	0.020
CRP (mg/L)	5.3 (2.2-33.2)	3.0 (2.3-4.8)	0.106
Iron (µmol/L)	13.8 (10.8-15.8)	13.6 (10.6-15.8)	0.443
UIBC (µmol/L)	38.3 (± 15)	49.1 (± 16)	< 0.001
TIBC (µmol/L)	51.4 (± 15)	63.8 (59.4-66.2)	< 0.001
Ferritin (µg/L)	250 (130-485)	109 (78-140)	0.002
Transferrin (g/L)	2.3 (± 0.8)	2.9 (± 0.7)	< 0.001
Transferrin saturation (%)	24 (20-27)	21 (18-26)	0.334
CEA (µg/L)	N/A	2.9 (2.6-5.3)	N/A
CA19-9 (kIU/L)	N/A	11.4 (8.3-18.3)	N/A
IL-6 (ng/L)	N/A	9.1 (5.8-15.6)	N/A
Hemoglobin (g/L)	137 (118-153)	126 (114-138)	0.022
Results are presented as median (25th-75th percentile) and as mean and standard deviation. Data were compared using the Mann-Whitney U test for non-normally distributed data and student t-test for normally distributed data. CRC - colorectal cancer group. CA19-9 - carbohydrate antigen 19-9. CEA - carcinoembryonic antigen. CRP - C-reactive protein. IL-6 - interleukin-6. N/A - not applicable. TIBC - total iron binding capacity. UIBC - unsaturated iron binding capacity. P < 0.05 was considered statistically significant.			

In the control group, the strongest positive correlation was observed between Hep and ferritin (rho = 0.84 (P < 0.001)). Total iron binding capacity, transferrin and UIBC exhibited moderate negative correlation with ferritin (rho = - 0.72, - 0.72 and - 0.66 respectively, all P < 0.001). Iron showed a weak negative correlation with Hep in the control group (rho = - 0.38 (P = 0.004)). Hepcidin showed a moderate positive correlation with CRP in the control group (rho = 0.52 (P < 0.001)), while in the CRC group the correlation was weaker (rho = 0.26 (P = 0.022)). In the CRC group, Hep was moderately positively correlated with ferritin (rho = 0.62 (P < 0.001)), weakly negatively correlated with transferrin (rho = - 0.43 (P < 0.001)), TIBC ((rho = - 0.37 (P < 0.001)) and UIBC ((rho = - 0.45 (P < 0.001)), and weakly positively correlated with CRP ((rho = 0.26 (P = 0.022)). All correlation coefficients are shown in Table 2.

**Table 2:** Correlation of hepcidin-25 with selected parameters of iron metabolism, inflammation (CRP) and tumor markers in colorectal cancer and control groups

	**CRC** **N = 82**	**Control group** **N = 58**
	Hepcidin	
	Correlation coefficient (P)	Correlation coefficient (P)
Iron	0.22 (0.045)	- 0.38 (0.004)
UIBC	- 0.45 (< 0.001)	- 0.66 (< 0.001)
TIBC	-0.37 (< 0.001)	- 0.724 (< 0.001)
Ferritin	0.62 (< 0.001)	0.84 (< 0.001)
Transferrin	- 0.43 (< 0.001)	- 0.72 (< 0.001)
Transferrin saturation	0.37 (< 0.001)	- 0.09 (0.53)
CRP	0.26 (0.022)	0.52 (< 0.001)
CEA	0.16 (0.270)	N/A
CA19-9	0.10 (0.470)	N/A
IL-6	0.30 (0.033)	N/A
Hemoglobin	0.15 (0.180)	- 0.17 (0.248)
Correlation data are presented as Spearman’s rho coefficients for non-normally distributed data and Pearson r coefficients for normally distributed data. CA19-9 - carbohydrate antigen 19-9. CRC - colorectal cancer group. CEA - carcinoembryonic antigen. CRP - C-reactive protein. IL-6 - interleukin-6. N/A - not applicable. TIBC - total iron binding capacity. UIBC - unsaturated iron binding capacity. P < 0.05 was considered statistically significant.		

In the CRC group, median Hep concentrations were lower in advanced stages, with the lowest concentrations observed in stage T3 (4.00 ng/mL (2.8-9.0). A statistically significant difference in hepcidin concentration was found between stage T0 and T3 (P = 0.043). These findings are summarized in Table 3. Tumor markers (CEA and CA19-9) did not exceed the established cut-off values.

**Table 3:** TNM staging of colorectal cancer and hepcidin serum concentrations

**TNM**	**N of subjects with CRC**	**Hepcidin (ng/mL)**	**P**
T0	9	33.0 (13.8-42.0)	0.043*
T1	3	48.3 (33.2-120.2)	
T2	14	12.8 (7.2-36.7)	
T3	38	4.0 (2.8-9.0)	
Tx	18	18.1 (5.4-30.4)	
Hepcidin concentrations are presented as median (25th-75th percentile). CRC - colorectal cancer group. TNM - tumor node metastasis. *T0 *vs.* T3 (Kruskal-Wallis test, P = 0.001). P < 0.05 was considered statistically significant.			

## Discussion

Our findings indicate altered iron metabolism in patients with sporadic CRC and a multifactorial alteration in iron metabolism in CRC. The significantly lower serum Hep concentrations between the CRC and control group further supports this observation. Although Hep concentrations varied considerably with both groups, the control group exhibited a higher median value and a wider interquartile range (25th-75th percentile) than the CRC group.

To the best of our knowledge, this is the first study to specifically evaluate serum Hep concentrations in patients with sporadic CRC, compare them to a control group and examine Hep as an iron-related marker in relation to iron metabolism analytes, inflammation marker and tumor markers. Additionally, this is the first study to analyze Hep concentrations by tumor stage.

Iron plays a crucial role in carcinogenesis, and CRC is particularly notable in the context of intestinal iron absorption (*10*). Patients diagnosed with CRC often present with anemia (*17*). The inflammatory cytokine IL-6 triggers the production of Hep, which inhibits erythropoiesis, leading to anemia (*18*). Interestingly, in our study, IL-6 showed a minimal correlation with Hep. However, concentration was above the cut off (9.1 ng/L; 25th-75th percentile: 5.8-15.6) suggesting the role of inflammation in CRC. C-reactive protein showed a moderate positive correlation with Hep in the control group and a weak correlation in the CRC group. This suggests that factors beyond inflammation, such as occult blood loss, may contribute to anemia in CRC. Our study revealed significant correlations between iron metabolism parameters and Hep, supporting the multifactorial nature of altered iron metabolism in cancer. A limited number of studies have investigated the role of Hep in CRC, primarily using cell lines (*11*, *19*). For instance, increased expression of Hep and ferritin heavy chain has been reported in CRC biopsies (*11*). Although comparison with cell lines studies is not possible, in some way this aligns with our findings of significantly different Hep and ferritin concentrations between CRC and control groups.

Further, the correlations between Hep and iron parameters were statistically significant, they were generally weak, but a notable trend in intergroup differences was observed. Particularly, the moderate correlation between Hep and ferritin is consistent with previous reports (*20*).

While ferritin has been explored as a tumor marker in several cancers, no consensus exists due to methodological variability (*13*, *21*, *22*). Some studies, such as Ramirez Cormona *et al.*, reported lower ferritin concentrations in CRC patients, a finding supported by our results (*22*). Di Grazia *et al.* observed an association between high Hep concentrations and poor prognosis in metastatic CRC (*23*). Since we had a non-metastatic CRC cohort, these data are not comparable, but it is important to note that our results showed lower Hep concentrations with advancing tumor stage, particularly in T3, indicating a possible link between tumor progression and Hep suppression. Our data showed that the main difference in Hep concentrations was between the earliest (T0) and more advanced (T3) tumor stages, with T3 showing notably lower serum hepcidin concentrations. Lower Hep concentrations observed in advanced tumor stages may reflect dysregulated iron metabolism and a potential role for Hep in advancing disease.

Hepcidin is abnormally secreted by CRC cells, promoting cancer growth and survival (*22*). Disrupted local hepcidin-ferroportin signaling is crucial for cancer growth, as alterations in this pathway contribute to cancer cell proliferation (*24*). The local production of Hep by cancer cells suggests that targeting this process could serve as a potential therapeutic strategy in cancer treatment. While all cells require iron for metabolism, cancer cells exhibit a heightened demand for iron due to its essential role in synthesizing deoxyribonucleotides for DNA replication and sustaining rapid proliferation (*25*). The iron-dependent enzyme ribonucleotide reductase catalyzes the rate-limiting step in DNA synthesis, making intracellular iron availability essential for enhanced cellular proliferation and DNA replication. Cancer cells demonstrate an increased ability to absorb iron, reduce iron export and bypass ferroptosis (iron-depended cell death), distinguishing them from non-malignant cells (*10*, *26*). This ability to evade ferroptosis contributes to their resistance to chemotherapy (*10*). Overall, higher Hep concentrations have been observed in various carcinomas, including CRC (*19*, *23*, *27*). In our study we report lower serum Hep concentrations in advanced stages of CRC. The results indicate that in the advanced stages of CRC, Hep production may be reduced. Such reduction likely allows undisturbed iron absorption, creating favorable conditions for cancer progression. A potential reason for discrepancies with previous findings may be differences in study design, particularly the use of cell lines or including metastatic CRC in other studies, whereas our cohort included patients with sporadic CRC.

Some studies have found no significant differences in Hep concentrations or iron metabolism parameters between CRC patients and controls, while others have observed slightly decreased Hep concentrations (*18*, *28*, *29*). Our findings suggest significant differences, but it is important to note that not all parameters are statistically significant. Differences in methods used to assess iron metabolism parameters are also important factors to consider when interpreting contradictory results. The analytical methods applied in our study have not been used in any previous research to our knowledge.

However, the present study has several limitations, including a relatively small sample size, particularly the low number of the female participants in the control group, lack of stratification by sex and age, especially among anemic patients, and no gender-based stratification for iron parameters. The immunoassay method used to detect Hep has limitations such as cross-reactivity between different Hep isoforms and high intra- and inter-individual variability. Additionally, the low number of female controls makes comparisons difficult. Nevertheless, hemoglobin concentration in the control group were above anemia cut off values, regardless of sex. It would be beneficial to increase the sample size to validate these findings and further explore the potential value of Hep as a biomarker for monitoring and managing colorectal cancer. Ideally, controls should include patients with positive occult blood test but no cancer. Furthermore, other conditions such as autoimmune and kidney diseases which can affect Hep expression, were not assessed in either group, which may have impacted the results. Finally, the study lacked power analysis, and the sample was underpowered within individual T-stage subgroups, especially T1 and T2. Nevertheless, the nature of a prospective cohort study, patients were selected independently of staging criteria.

In conclusion, our results suggest that Hep concentrations are lower in advanced stages of sporadic, non-metastatic CRC and could serve as a valuable biomarker reflecting tumor stage. Hepcidin shows a moderate correlation with ferritin and like ferritin, may have potential as a tumor marker in the future research. The observed pattern of lower Hep concentrations across tumor stages may also reveal a potential relationship between tumor stage and Hep regulation, which could have implications for understanding disease and identifying new therapeutic approaches. Evaluating Hep as a potential therapeutic target in CRC represents an important direction for future research.

## Data Availability

All data generated and analyzed in the presented study are included in this published article.

## References

[1] Charlebois E Pantopoulos K. Nutritional Aspects of Iron in Health and Disease. Nutrients. 2023;15:2441. 10.3390/nu15112441 37299408 PMC10254751

[2] Ganz T Nemeth E. The hepcidin-ferroportin system as a therapeutic target in anemias and iron overload disorders. Hematology (Am Soc Hematol Educ Program). 2011;2011:538–42. 10.1182/asheducation-2011.1.538 22160086 PMC4034574

[3] Camaschella C Nai A Silvestri L. Iron metabolism and iron disorders revisited in the hepcidin era. Haematologica. 2020;105:260–72. 10.3324/haematol.2019.232124 31949017 PMC7012465

[4] Nemeth E Ganz T. Hepcidin and Iron in Health and Disease. Annu Rev Med. 2023;74:261–77. 10.1146/annurev-med-043021-032816 35905974 PMC9943683

[5] Teh MR Armitage AE Drakesmith H. Why cells need iron: a compendium of iron utilisation. Trends Endocrinol Metab. 2024;35:1026–49. 10.1016/j.tem.2024.04.015 38760200 PMC11616622

[6] Yiannikourides A Latunde-Dada GO. A Short Review of Iron Metabolism and Pathophysiology of Iron Disorders. Medicines (Basel). 2019;6:85. 10.3390/medicines6030085 31387234 PMC6789448

[7] Ward DG Roberts K Brookes MJ Joy H Martin A Ismail T Increased hepcidin expression in colorectal carcinogenesis. World J Gastroenterol. 2008;14:1339–45. 10.3748/wjg.14.1339 18322945 PMC2693679

[8] Diepeveen LE Laarakkers CMM Martos G Pawlak ME Uguz FF Verberne K Provisional standardization of hepcidin assays: creating a traceability chain with a primary reference material, candidate reference method and a commutable secondary reference material. Clin Chem Lab Med. 2019;57:864–72. 10.1515/cclm-2018-0783 30485171

[9] Huang Y Cao D Chen Z Chen B Li J Wang R Iron intake and multiple health outcomes: Umbrella review. Crit Rev Food Sci Nutr. 2023;63:2910–27. 10.1080/10408398.2021.1982861 34583608

[10] Estêvão D da Cruz-Ribeiro M Cardoso AP Costa AM Oliveira MJ Duarte TL Iron metabolism in colorectal cancer: a balancing act. Cell Oncol (Dordr). 2023;46:1545–58. 10.1007/s13402-023-00828-3 37273145 PMC10698107

[11] Sornjai W Nguyen Van Long F Pion N Pasquer A Saurin JC Marcel V Iron and hepcidin mediate human colorectal cancer cell growth. Chem Biol Interact. 2020;319:109021. 10.1016/j.cbi.2020.109021 32092301

[12] World Health Organization. GLOBOCAN Cancer Today Data 2022. Available from: https://gco.iarc.fr/today/home. Accessed December 6th, 2024.

[13] Gao Y Wang J Zhou Y Sheng S Qian SY Huo X. Evaluation of Serum CEA, CA19-9, CA72-4, CA125 and Ferritin as Diagnostic Markers and Factors of Clinical Parameters for Colorectal Cancer. Sci Rep. 2018;8:2732. 10.1038/s41598-018-21048-y 29426902 PMC5807317

[14] Banjari I Hjartaker A. Dietary sources of iron and vitamin B12: Is this the missing link in colorectal carcinogenesis? Med Hypotheses. 2018;116:105–10. 10.1016/j.mehy.2018.05.003 29857891

[15] Simundic AM Bolenius K Cadamuro J Church S Cornes MP van Dongen-Lases EC Joint EFLM-COLABIOCLI Recommendation for venous blood sampling. Clin Chem Lab Med. 2018;56:2015–38. 10.1515/cclm-2018-0602 30004902

[16] Rolic T Mandic S Lukic I Horvat V Banjari I. Verification of the Automated ELISA Assay for Hepcidin-25 in Human Serum. SEEMEJED. 2023;7:36–41. 10.26332/seemedj.v7i1.266

[17] Gvirtzman R Livovsky DM Tahover E Goldin E Koslowsky B. Anemia can predict the prognosis of colorectal cancer in the pre-operative stage: a retrospective analysis. World J Surg Oncol. 2021;19:341. 10.1186/s12957-021-02452-7 34876136 PMC8653538

[18] Weiss G Ganz T Goodnough LT. Anemia of inflammation. Blood. 2019;133:40–50. 10.1182/blood-2018-06-856500 30401705 PMC6536698

[19] Pusatcioglu CK Nemeth E Fantuzzi G Llor X Freels S Tussing-Humphreys L Systemic and tumor level iron regulation in men with colorectal cancer: a case control study. Nutr Metab (Lond). 2014;11:21. 10.1186/1743-7075-11-21 24872837 PMC4037273

[20] Galetti V Stoffel NU Sieber C Zeder C Moretti D Zimmermann MB. Threshold ferritin and hepcidin concentrations indicating early iron deficiency in young women based on upregulation of iron absorption. EClinicalMedicine. 2021;39:101052. 10.1016/j.eclinm.2021.101052 34401687 PMC8350021

[21] Gackowski D Kruszewsk M Banaszkiewicz Z Jawien A Olinski R. Lymphocyte labile iron pool, plasma iron, transferrin saturation and ferritin levels in colon cancer patients. Acta Biochim Pol. 2002;49:269–72. 10.18388/abp.2002_3845 12136950

[22] Ramírez-Carmona W Diaz-Fabregat B Yuri Yoshigae A Musa de Aquino A Scarano WR de Souza Castilho AC Are Serum Ferritin Levels a Reliable Cancer Biomarker? A Systematic Review and Meta-Analysis. Nutr Cancer. 2022;74:1917–26. 10.1080/01635581.2021.1982996 34607491

[23] Di Grazia A Di Fusco D Franze E Colella M Strimpakos G Salvatori S Hepcidin Upregulation in Colorectal Cancer Associates with Accumulation of Regulatory Macrophages and Epithelial-Mesenchymal Transition and Correlates with Progression of the Disease. Cancers (Basel). 2022;14: 10.3390/cancers14215294 36358713 PMC9658525

[24] Schwartz AJ Goyert JW Solanki S Kerk SA Chen B Castillo C Hepcidin sequesters iron to sustain nucleotide metabolism and mitochondrial function in colorectal cancer epithelial cells. Nat Metab. 2021;3:969–82. 10.1038/s42255-021-00406-7 34155415 PMC8316354

[25] Torti SV Manz DH Paul BT Blanchette-Farra N Torti FM. Iron and Cancer. Annu Rev Nutr. 2018;38:97–125. 10.1146/annurev-nutr-082117-051732 30130469 PMC8118195

[26] Aksan A Farrag K Aksan S Schroeder O Stein J. Flipside of the Coin: Iron Deficiency and Colorectal Cancer. Front Immunol. 2021;12:635899. 10.3389/fimmu.2021.635899 33777027 PMC7991591

[27] Lin F Tuffour A Hao G Peprah FA Huang A Zhou Y Distinctive modulation of hepcidin in cancer and its therapeutic relevance. Front Oncol. 2023;13:1141603. 10.3389/fonc.2023.1141603 36895478 PMC9989193

[28] Cao H Wang C Chai R Dong Q Tu S. Iron intake, serum iron indices and risk of colorectal adenomas: a meta-analysis of observational studies. Eur J Cancer Care (Engl). 2017;26: 10.1111/ecc.12486 26956572

[29] Phillips E Horniblow RD Poole V Bedford M Ward DG Kirkham AJ A potential role for hepcidin in obesity-driven colorectal tumorigenesis. Oncol Rep. 2018;39:392–400. 10.3892/or.2017.6062 29115635

